# Laparoscopic versus open nephroureterectomy to treat localized and/or locally advanced upper tract urothelial carcinoma: oncological outcomes from a multicenter study

**DOI:** 10.1186/s12893-016-0202-x

**Published:** 2017-01-17

**Authors:** Jian-Ye Liu, Ying-Bo Dai, Fang-Jian Zhou, Zhi Long, Yong-Hong Li, Dan Xie, Bin Liu, Jin Tang, Jing Tan, Kun Yao, Le-Ye He

**Affiliations:** 1 Department of Urology, The Third Xiangya Hospital of Central South University, No.138, Tongzipo Road, Changsha, 410013 Hunan China; 2 Institute of Prostate Disease of Central South University, No.138, Tongzipo Road, Changsha, 410013 Hunan China; 3 Department of Urology, Sun Yat-sen University Cancer Center, No. 651, Dongfeng Road East, Guangzhou, 510060 Guangdong China; 4 Department of Pathology, Sun Yat-sen University Cancer Center, No. 651, Dongfeng Road East, Guangzhou, 510060 Guangdong China; 5 State Key Laboratory of Oncology in South China, No. 651, Dongfeng Road East, Guangzhou, 510060 Guangdong China

**Keywords:** Upper tract urothelial carcinoma, Laparoscopic radical nephroureterectomy, Open radical nephroureterectomy, Recurrent, Survival, Oncological, Outcomes

## Abstract

**Background:**

Many studies have reported the oncological outcomes between open radical nephroureterectomy (ONU) and laparoscopic radical nephroureterectomy (LNU) of upper tract urothelial carcinoma (UTUC). However, few data have focused on the oncological outcomes of LNU in the subgroup of localized and/or locally advanced UTUC (T_1–4_/N_0-X_). The purpose of this study was to compare the oncological outcomes of LNU *vs*. ONU for the treatment in patients with T_1–4_/N_0-X_ UTUC.

**Methods:**

We collected and analyzed the data and clinical outcomes retrospectively for 265 patients who underwent radical nephroureterectomy for T_1–4_/N_0-X_ UTUC between April 2000 and April 2013 at two Chinese tertiary hospitals. Survival was estimated using the Kaplan-Meier method. Cox’s proportional hazards model was used for univariate and multivariate analysis.

**Results:**

The mean patient age was 62.0 years and the median follow-up was 60.0 months. Of the 265 patients, 213 (80.4%) underwent conventional ONU, and 52 (19.6%) patients underwent LNU. The groups differed significantly in their presence of previous hydronephrosis, presence of previous bladder urothelial carcinoma, and management of distal ureter (*P* < 0.05). The predicted 5-year intravesical recurrence- free survival (RFS) (79% *v*s. 88%, *P* = 0.204), overall RFS (47% *v*s. 59%, *P* = 0.076), cancer-specific survival (CSS) (63% *v*s. 70%, *P* = 0.186), and overall survival (OS) (61% *v*s. 55%, *P* = 0.908) rates did not differ between the ONU and LNU groups. Multivariable Cox proportional regression analysis showed that surgical approach was not significantly associated with intravesical RFS (odds ratio [OR] 1.23, 95% confidence interval [CI] 0.46–3.65, *P* = 0.622), Overall RFS (OR 0.99, 95% CI 0.54–1.83, *P* = 0.974), CSS (OR 1.38, 95% CI 0.616–3.13, *P* = 0.444), or OS (OR 1.61, 95% CI 0.81–3.17, *P* = 0.17).

**Conclusions:**

The results of this retrospective study showed no statistically significant differences in intravesical RFS, overall RFS, CSS, or OS between the laparoscopy and the open groups. Thus, LNU can be an alternative to the open procedure for T_1–4_/N_0-X_ UTUC. Further studies, including a multi-institutional, prospective study are required to confirm these findings.

## Background

Upper urinary tract urothelial carcinoma (UTUC) is a relatively rare malignancy. It is estimated to comprise 10% of all renal tumors and 5% of urothelial carcinomas overall [1]. Open radical nephroureterectomy (ONU), with excision of the ipsilateral bladder cuff, is the standard treatment for UTUC [2, 3]. However, laparoscopic radical nephroureterectomy (LNU), first performed by Clayman et al. in 1991, has emerged as an accepted minimally invasive treatment alternative to ONU [4]. Subsequently, there have been numerous retrospective reports comparing the oncological outcomes between ONU and LNU [5–18] and one prospective series [19]. To date, none of the studies have shown a significant difference between the techniques in terms of overall survival (OS), recurrence-free survival (RFS), and cancer-specific survival (CSS). Only one study showed that there was a trend toward an independent association between surgical approach and RFS [13], and three studies showed a higher risk of intravesical RFS with LNU [7, 20, 21]. However, these studies focused on the oncological outcomes among the entire cohort of UTUC patients. Especially, they included a great many pTa stage and organ-confined UTUC. As experience with LNU grows, case selection has expanded to include more complex cases, resulting in carefully selected localized and/or locally advanced UTUC and larger tumors being operated on laparoscopically. However, until recently, only one study has focused on the oncological outcomes of LNU in the subgroup of localized and/or locally advanced UTUC [22]. Hence, the present study aimed to compare intravesical RFS, overall RFS, CSS, and OS between ONU and LNU for localized and/or locally advanced UTUC (T_1–4_/N_0-X_), performed in two Chinese tertiary teaching hospitals.

## Methods

### Patients

After institutional review board approval was obtained, a total of 265 consecutive patients, who were identified as having localized and/or locally advanced UTUC (T_1–4_/N_0-X_), and subsequently underwent ONU or LNU between April 2000 and April 2013 in The Third Xiangya Hospital of Central South University and the Sun Yat-sen University Cancer Center, were investigated in this study. Exclusion criteria were the presence of any known metastatic disease at the time of surgery, and radical cystectomy with concomitant radical nephroureterectomy (RNU). All patients had undergone computed tomography, and/or intravenous urography, and/or cystoscopy, and/or urine cytology. Diagnostic ureteroscopy with biopsies has been used to stage tumors accurately in some patients. In addition, none of the patients had received preoperative chemotherapy.

### Surgical procedures

Surgery was performed by surgeons according to the standard criteria for RNU. The ONU was performed as either a double-access incision: a loin incision and an iliac incision; or a midline incision was performed from the subxiphoid down to the pelvis. The kidney, Gerota fascia, perinephric fat, the entire length of ureter, and the bladder cuff were excised en bloc. Regional lymphadenectomy was generally performed if lymph nodes were abnormal on preoperative computed tomography or if they were palpable intra-operatively. Extended lymphadenectomy was not performed routinely. The LNU was performed using the retroperitoneal or transperitoneal approach. The range of resection was technically as the same as in the ONU. The patients were fully informed with regard to the surgical approach (laparoscopic *vs*. open surgery) and its possible complications, and the choice of choice of surgical procedure was nonrandomized; it depended on patient and surgeon preference and experience. In the laparoscopic group, only one patient converted to open surgery. Distal ureter management approaches were categorized as follows: (1) extravesical ureter; (2) open intravesical; and (3) endoscopic.

### Pathological and clinical evaluation

All surgical specimens were processed according to standard pathological procedures and anatomical pathologists at two institutions reviewed all slides. Centralized pathological review and reclassification of specimens was not performed. Tumors were staged according to the 2002 American Joint Committee on Cancer TNM classification system, and graded according to the 2004 World Health Organization/International Society of Urologic Pathology (WHO/ISUP) consensus classification. The tumor site was defined as renal pelvis, ureter, or both renal pelvis and ureter. Tumor multifocality was defined as the synchronous presence of two or more pathologically confirmed tumors in any upper urinary tract location (renal pelvis or ureter). Lymphovascular invasion (LVI) was defined as the unequivocal presence of tumor cells within an endothelium-lined space, with no underlying muscular walls.

### Follow-up regimen

Patients were generally followed-up every 3 months for 2 years after RNU, every 6 months for the next 3 years, and annually thereafter. Patients’ histories were taken, and they underwent a physical examination, routine blood evaluation, urinary cytology, chest radiography, cystoscopic evaluation of the bladder, and radiographic evaluation of the contralateral upper urinary tract at each visit. Elective bone scans, computerized tomography, or magnetic resonance imaging were performed when indicated clinically.

### Statistical analysis

Statistical analyses were performed using the statistical software SPSS version 16.0 (SPSS, Inc., Chicago, IL). We compared the clinical and pathological characteristics of the two surgical technique groups (ONU *v*s. LNU) using Student’s t-test for continuous variables and the chi-squared test for categorical variables. The primary end-points were intravesical RFS, overall RFS, CSS, and OS. Intravesical recurrences included recurrences within the bladder only. Overall recurrent disease included recurrences within the bladder, as well as contralateral recurrences, tumor relapse in the operative field, regional lymph nodes, port site metastasis, and/or distant metastasis. CSS was defined as the time interval between the date of RNU and the end point, including death or censoring. We defined “OS time” as the period between the date of the first operation for the original disease and the date of patient death (from any cause). Survival probabilities were estimated using the Kaplan-Meier method, and the log-rank test was applied to compare survival curves. Univariate and multivariate Cox proportional hazards regression analyses were performed to determine the association between surgical approach and clinical outcomes. All reported *P*-values were two-sided, and statistical significance was set at *P* ≤ 0.05.

## Results

### Characteristics of patients

The study cohort comprised 265 assessable patients. The ONU was performed in 213 (80.4%) *v*s. LNU in 52 (19.6%) patients. The clinical and pathological details for each of the groups are presented in Table 1. The open surgery preferred the presence of previous hydronephrosis, absence of previous bladder urothelial carcinoma, and underwent extravesical management of the distal ureter (*P* < 0.05 for all) (Table 1).

**Table 1:** Clinical and pathological characteristics of 265 patients treated with either ONU or LNU for UTUC

Clinical or pathological characteristic	Total cases (*n* = 265)	Type of procedure		*P* value
		ONU (*n* = 213)	LNU (*n* = 52)	
Mean age (SD), years	62.0 (10.7)	62.5 (10.7)	60.2 (10.7)	0.167^a^
Gender, n (%)				0.445^b^
Male	198 (74.7)	157 (73.7)	41 (78.8)	
Female	67 (25.3)	56 (26.3)	11 (21.2)	
Smoking, n (%)				0.207^b^
No	158 (59.6)	131 (61.5)	27 (51.9)	
Yes	107 (40.4)	82 (38.5)	25 (48.1)	
Previous hydronephrosis, n (%)				**0.032** ^**b**^
No	89 (33.6)	65 (30.5)	24 (46.2)	
Yes	176 (66.4)	148 (69.5)	28 (53.8)	
Previous bladder urothelial carcinoma, n (%)				**<0.001** ^**b**^
No	225 (84.9)	201 (94.4)	24 (46.2)	
Yes	40 (15.1)	12 (5.6)	28 (53.8)	
Concomitant bladder urothelial carcinoma, n (%)				0.274^b^
No	221 (83.4)	175 (82.2)	46 (88.5)	
Yes	44 (16.6)	38 (17.8)	6 (11.5)	
Laterality, n (%)				0.598^b^
Left	134 (50.6)	106 (49.8)	28 (53.8)	
Right	131 (49.4)	107 (50.2)	24 (46.2)	
Tumor location, n (%)				0.070^b^
Renal pelvis	119 (44.9)	89 (41.8)	30 (57.7)	
Ureter	129 (48.7)	108 (50.7)	21 (40.4)	
Renal pelvis and ureter	17 (6.4)	16 (7.5)	1 (1.9)	
Mean tumor size (SD), cm	3.8 (2.0)	3.9 (2.1)	3.3 (1.4)	0.076^a^
Tumor focality, n (%)				0.453^b^
Unifocal	156 (58.9)	123 (57.7)	33 (63.5)	
Multifocal	109 (41.1)	90 (42.3)	19 (36.5)	
LVI, n(%)				0.964^b^
No	193 (72.8)	155 (72.8)	38 (73.1)	
Yes	72 (27.2)	58 (27.2)	14 (26.9)	
Tumor grade, n (%)				0.570^b^
Low	103 (38.9)	81 (38.0)	22 (42.3)	
High	162 (61.1)	132 (62.0)	30 (57.7)	
pT stage, n (%)				0.546^b^
pT1	85 (32.1)	65 (30.5)	20 (38.5)	
pT2	56 (21.1)	46 (21.6)	10 (19.2)	
pT3/pT4	124 (46.8)	102 (47.9)	22 (42.3)	
pN stage, n (%)				0.613^b^
pN0	109 (41.1)	86 (40.4)	23 (44.2)	
pNx	156 (58.9)	127 (59.6)	29 (55.8)	
Distal ureter management, n (%)				**<0.001** ^**b**^
Extravesical	58 (21.9)	55 (25.8)	3 (8.9)	
Open intravesical	154 (58.1)	139 (65.3)	15 (28.8)	
Endoscopic	53 (20.0)	19 (8.9)	34 (65.4)	
Adjuvant chemotherapy, n (%)				0.945^b^
No	208 (78.5)	167 (78.4)	41 (78.8)	
Yes	57 (21.5)	46 (21.6)	11 (21.2)	

Bold values indicate that *P*-value ≤ 0.05, and considered statistically significant. *ONU* open radical nephroureterectomy, *LNU* laparoscopic radical nephroureterectomy, *UTUC* upper tract urothelial carcinoma, *LVI* lymphovascular invasion, *SD* standard deviation

ªstudent’s test

^b^chi-square test

### Survival analysis

**Fig. 1:**
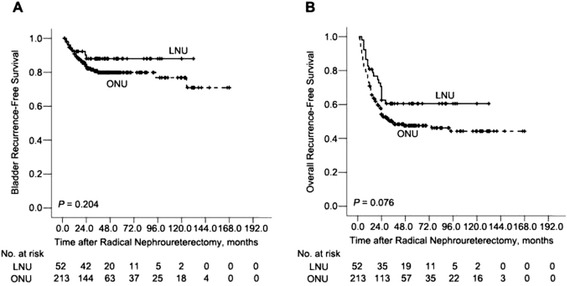
Intravesical recurrence-free survival (**a**) and Overall recurrence-free survival rates (**b**) in 265 patients treated with either ONU (*n* = 213) or LNU (*n* = 52) for UTUC

**Fig. 2:**
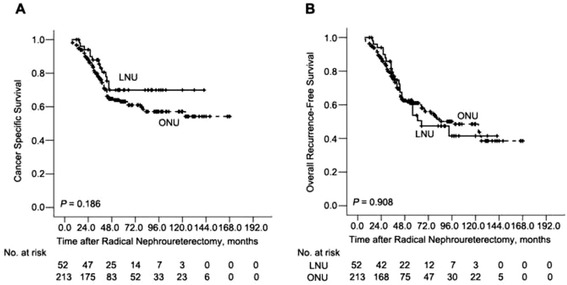
Cancer-specific survival (**a**) and Overall survival rates (**b**) in 265 patients treated with either ONU (*n* = 213) or LNU (*n* = 52) for UTUC

At last follow-up, there were 46 (17.4%) bladder recurrences, including 40 (18.8%) in the ONU group and six (11.5%) in the LNU group. The 5-year intravesical RFS estimates for the ONU and LNU groups were 79% and 88%, respectively (*P* = 0.204) (Fig. 1a). The total number of recurrence in the ONU and LNU groups were 109 (51.1%) and 20 (38.5%), respectively. Overall RFS for the ONU and LNU groups at 5 years were 47% and 59%, respectively (*P* = 0.076) (Fig. 1b). In all, 84 patients (31.7%) patients suffered disease progression and metastasis during the study period, including 71 (33.3%) in the ONU group and 13 (25.0%) in the LNU group. Estimated 5-year CSS estimates for ONU and LNU groups were 63% and 70%, respectively, which was non-significant (*P* = 0.186) (Fig. 2a). In the open group, 84 (39.4%) patients died (from any cause). The 5-year OS rate was 61%. Death occurred (from any cause) in 23 patients (44.2%) in the laparoscopy group. The 5-year OS rate was 55%. No statistically significant difference was found for the OS rate between the two groups (*P* = 0.908) (Fig. 2b).

Predictors of higher intravesical RFS rate on multivariate analysis included concomitant bladder urothelial carcinoma (odds ratio [OR] 2.71, 95% confidence interval [CI] 1.22–5.99, *P* = 0.014), undergoing extravesical management of distal ureter (*P* < 0.001), and not having received adjuvant chemotherapy (OR 0.28, 95% CI 0.09–0.90, *P* = 0.033) (Table 2). However, there was no association between surgical approach and intravesical RFS in multivariate cox regression (OR 1.23, 95% CI 0.46–3.65, *P* = 0.622) (Table 2).

**Table 2:** Univariable and multivariable cox regression models predicting intravesical RFS and Overall RFS of 265 patients with UTUC after radical nephroureterectomy

Variable	Intravesical RFS				Overall RFS			
	Univariate analysis		Multivariate analysis		Univariate analysis		Multivariate analysis	
	OR (95% CI)	*P* value	OR (95% CI)	*P* value	OR (95% CI)	*P* value	OR (95% CI)	*P* value
Age, continuous	0.99 (0.96–1.01)	0.359	0.98 (0.96–1.01)	0.239	1.00 (0.99–1.01)	0.981	0.99 (0.97–1.01)	0.232
Gender								
Male	1	0.181	1	0.07	1	0.94	1	0.258
Female	0.59 (0.28–1.3)		0.45 (0.19–1.07)		1.02 (0.69–1.50)		0.77 (0.48–1.22)	
Smoking								
No	1	0.273	1	0.357	1	**0.021**	1	0.057
Yes	1.41 (0.76–2.58)		1.41 (0.68–2.89)		1.54 (0.07–2.22)		1.52 (0.99–2.33)	
Previous hydronephrosis								
No	1	0.768	1	0.269	1	0.08	1	0.83
Yes	1.10 (0.59–2.03)		0.65 (0.30–1.40)		1.41 (0.96–2.08)		0.94 (0.56–1.59)	
Previous bladder urothelial carcinoma								
No	1	0.394	1	0.491	1	0.8	1	0.291
Yes	1.56 (0.56–4.7)		0.65 (0.19–2.24)		1.10 (0.52–2.37)		1.60 (0.67–3.84)	
Concomitant bladder urothelial carcinoma								
No	1	**0.048**	1	**0.014**	1	0.369	1	0.585
Yes	1.95 (1.01–3.76)		2.71 (1.22–5.99)		1.23 (1.79–1.91)		1.15 (0.70–1.89)	
Laterality								
Left	1	0.276	1	0.573	1	0.789	1	0.534
Right	1.38 (0.77–2.48)		1.20 (0.64–2.24)		0.95 (0.68–1.35)		1.13 (0.78–1.64)	
Tumor location								
Renal pelvis	1	0.093	1	0.129	1	**0.001**	1	**0.003**
Ureter	1.67 (0.89–3.17)		1.57 (0.65–3.78)		1.25 (0.86–1.80)		1.60 (0.96–2.67)	
Renal pelvis and ureter	2.82 (1.02–7.78)		3.72 (1.04–13.25)		3.15 (1.74–5.71)		3.52 (1.70–7.26)	
Tumor size, continuous	0.90 (0.76–1.06)	0.209	0.91 (0.74–1.11)	0.353	1.07 (0.98–1.17)	0.112	1.02 (0.92–1.13)	0.682
Tumor focality								
Unifocal	1	0.608	1	0.757	1	**0.015**	1	0.492
Multifocal	1.17 (0.65–2.09)		1.12 (0.56–2.23)		1.54 (1.09–2.17)		1.15 (0.77–1.72)	
LVI								
No	1	0.582	1	0.746	1	*<* **0.001**	1	*<* **0.001**
Yes	0.83 (0.42–1.63)		0.89 (0.43–1.83)		2.20 (1.54–3.13)		2.03 (1.39–2.96)	
Tumor grade								
Low	1	0.91	1	0.525	1	*<* **0.001**	1	*<* **0.001**
High	0.97 (0.54–1.74)		0.79 (0.39–1.62)		3.72 (2.41–5.73)		2.47 (1.51–4.03)	
pT stage								
pT1	1	0.434	1	0.471	1	*<* **0.001**	1	*<* **0.001**
pT2	1.48 (0.70–3.10)		1.18 (0.52–2.67)		1.54 (0.86–2.76)		1.72 (1.06–3.03)	
pT3/pT4	0.96 (0.48–1.93)		1.67 (0.73–3.78)		3.46 (2.18–5.50)		2.57 (1.47–4.48)	
pN stage								
pN0	1	0.097	1	0.308	1	0.489	1	0.677
pNx	1.73 (0.91–3.28)		1.45 (0.71–2.94)		1.13 (0.79–1.62)		1.09 (0.74–1.61)	
Distal ureter management								
Extravesical	1	*<* **0.001**	1	*<* **0.001**	1	**0.011**	1	0.129
Open intravesical	0.32 (0.18–0.59)		0.25 (0.13–0.50)		0.63 (0.43–0.94)		0.73 (0.47–1.12)	
Endoscopic	0.17 (0.06–0.49)		0.11 (0.03–0.42)		0.46 (0.27–0.79)		0.52 (0.27–1.01)	
Adjuvant chemotherapy								
No	1	**0.043**	1	**0.033**	1	**0.035**	1	0.194
Yes	0.35 (0.12–0.97)		0.28 (0.09–0.90)		1.52 (1.03–2.26)		0.74 (0.46–1.17)	
Type of procedure								
ONU	1	0.211	1	0.622	1	0.082	1	0.974
LNU	0.58 (0.25–1.34)		1.23 (0.46–3.65)		0.66 (0.41–1.06)		0.99 (0.54–1.83)	

Bold values indicate that *P*-value ≤ 0.05, and considered statistically significant. *ONU* open radical nephroureterectomy, *LNU* laparoscopic radical nephroureterectomy, *UTUC* upper tract urothelial carcinoma, *LVI* lymphovascular invasion, *RFS* recurrence-free survival, *OR* odds ratio, *CI* confidence interval

Meanwhile, in the multivariate analysis, the type of surgery was not an independent predictor of overall RFS (OR 0.99, 95% CI 0.54–1.83, *P* = 0.974) (Table 2). However, four clinical pathological parameters were identified as probable predictors of overall RFS in multivariate Cox regression models: tumor location (*P* = 0.003), LVI (OR 2.03, 95% CI 1.39–2.96, *P* < 0.001), tumor grade (OR 2.47, 95% CI 1.51–4.03, *P* < 0.001), and pT stage (*P* < 0.001) (Table 2).

Table 3 presents the results of the multivariate analysis examining predictors of CSS and OS in the cohort. On multivariate analysis, LVI, tumor grade, and pT stage were the only independent predictors of CSS (*P* < 0.05 for all; Table 3). Similarly, LVI, tumor grade, and pT stage were the independent predictors of OS (*P* < 0.05 for all; Table 3). The type of procedure, ONU or LNU, was not an independent predictor of CSS (OR 1.38, 95% CI 0.61–3.13, *P* = 0.444; Table 3) or OS (OR 1.61, 95% CI 0.82–3.17, *P* = 0.17; Table 3).

**Table 3:** Univariable and multivariable cox regression models predicting CSS and OS of 265 patients with UTUC after radical nephroureterectomy

Variable	CSS				OS			
	Univariate analysis		Multivariate analysis		Univariate analysis		Multivariate analysis	
	OR (95% CI)	*P* value	OR (95% CI)	*P* value	OR (95% CI)	*P* value	OR (95% CI)	*P* value
Age, continuous	1.01 (0.99–1.03)	0.282	0.99 (0.97–1.02)	0.641	1.03 (1.01–1.04)	**0.011**	1.02 (0.99–1.04)	0.159
Gender								
Male	1	0.681	1	0.93	1	0.827	1	0.66
Female	1.11 (0.68–1.80)		1.03 (0.56–1.88)		1.05 (0.67–1.64)		0.89 (0.53–1.50)	
Smoking								
No	1	0.171	1	0.302	1	0.113	1	0.119
Yes	1.37 (0.87–2.14)		1.34 (0.77–2.36)		1.38 (0.93–2.04)		1.46 (0.91–2.34)	
Previous hydronephrosis								
No	1	0.115	1	0.439	1	0.058	1	0.149
Yes	1.47 (0.91–2.38)		1.30 (0.67–2.50)		1.51 (0.99–2.32)		1.51(0.86–2.65)	
Previous bladder urothelial carcinoma								
No	1	0.606	1	0.833	1	0.926	1	0.966
Yes	1.24 (0.54–2.86)		1.23 (0.37–3.48)		0.96 (0.42–2.19)		1.02 (0.39–2.69)	
Concomitant bladder urothelial carcinoma								
No	1	0.636	1	0.712	1	0.48	1	0.411
Yes	1.14 (0.66–1.97)		1.13 (0.60–2.14)		0.83 (0.49–1.41)		0.77 (0.42–1.43)	
Laterality								
Left	1	0.512	1	0.11	1	0.772	1	0.23
Right	0.87 (0.56–1.33)		1.50 (0.91–2.45)		0.95 (0.65–1.38)		1.30 (0.85–1.99)	
Tumor location								
Renal pelvis	1	**0.029**		0.07	1	0.4	1	0.458
Ureter	1.35 (0.85–2.15)		1.77 (0.93–3.37)		1.15 (0.77–1.72)		1.31 (0.76–2.25)	
Renal pelvis and ureter	2.61 (1.28–5.34)		2.69 (1.08–6.70)		1.62 (0.79–3.32)		1.63 (0.70–3.78)	
Tumor size, continuous	1.13 (1.03–1.26)	**0.015**	1.07 (0.95–1.21)	0.268	1.09 (0.99–1.19)	0.083	1.05 (0.94–1.18)	0.364
Tumor focality								
Unifocal	1	0.07	1	0.871	1	0.085	1	0.694
Multifocal	1.49 (0.97–2.28)		0.96 (0.58–1.60)		1.40 (0.96–2.04)		1.09 (0.70–1.70)	
LVI								
No	1	*<* **0.001**	1	**0.003**	1	*<* **0.001**	1	**0.002**
Yes	2.20 (1.54–3.13)		2.0 (1.26–3.17)		2.20 (1.50–3.23)		1.88 (1.25–2.83)	
Tumor grade								
Low	1	*<* **0.001**		*<* **0.001**	1	*<* **0.001**	1	**0.001**
High	11.23 (4.89–25.81)		6.95 (2.87–16.83)		4.03 (2.47–6.56)		2.59 (1.50–4.49)	
pT stage								
pT1	1	*<* **0.001**		**0.002**	1	*<* **0.001**	1	**0.004**
pT2	1.91 (0.75–4.84)		1.69 (1.11–2.59)		1.36 (0.69–2.70)		1.40 (0.91–2.14)	
pT3/pT4	7.40 (3.55–15.43)		2.83 (1.20–6.66)		4.12 (2.43–6.99)		2.29 (1.21–4.36)	
pN stage								
pN0	1	0.429	1	0.494	1	0.785	1	0.645
pNx	0.84 (0.55–1.29)		0.84 (0.51–1.38)		0.95 (0.64–1.40)		0.90 (0.59–1.39)	
Distal ureter management								
Extravesical	1	0.434	1	0.277	1	0.538	1	0.921
Open intravesical	1.09 (0.65–1.85)		1.35 (0.75–2.41)		0.77 (0.49–1.22)		0.93 (0.57–1.52)	
Endoscopic	0.73 (0.36–1.48)		0.74 (0.30–1.84)		0.87 (0.50–1.50)		0.86 (0.41–1.82)	
Adjuvant chemotherapy								
No	1	*<* **0.001**	1	0.111	1	*<* **0.001**	1	0.331
Yes	2.93 (1.88–4.56)		1.54 (0.90–2.63)		2.13 (1.40–3.22)		1.28 (0.78–2.09)	
Type of procedure								
ONU	1	0.191	1	0.444	1	0.909	1	0.17
LNU	0.67 (0.37–1.22)		1.38 (0.61–3.13)		1.03 (0.65–1.63)		1.61 (0.82–3.17)	

Bold values indicate that *P*-value ≤ 0.05, and considered statistically significant. *ONU* open radical nephroureterectomy, *LNU* laparoscopic radical nephroureterectomy, *UTUC* upper tract urothelial carcinoma, *LVI* lymphovascular invasion, *CSS c*ancer specific survival, *OS* overall survival, *OR* odds ratio, *CI* confidence interval

## Discussion

Clayman et al. performed the first successful LNU in 1991 [4]. Multiple reports have since described the efficacy of LNU for favorable-risk UTUC patients regarding cancer control [5–19]. In recent years, experienced surgeons have expanded their criteria for LNU for large or locally advanced UTUC, which indicated the effectiveness of laparoscopic surgery. To compare the efficacy of LNU and ONU in localized and/or locally advanced UTUC, we performed the present study, including 265 patients with T_1–4_/N_0-X_ UTUC (213 ONU *vs*. 52 LNU) treated with RNU. The Kaplan-Meier plot illustrated no significant difference in survival between the two groups of different procedures. Multivariate analysis suggested the equivalence of LNU and ONU in terms of intravesical RFS, overall RFS, CSS, and OS. During the later courses of our study, Kim et al. [22] reported that the 5-year OS and CSS rates were lower in the LNU group than in the ONU group in patients with locally advanced UTUC. Furthermore, on multivariable analysis, LNU was found to be an independent predictor of poorer OS and CSS than ONU. However, the study has some limitations: On the one hand, the cohort patients included N+ disease. On the other hand, the study did not analyze the cigarette smoking status, despite the fact that exposure to smoking is a significant risk factor for bladder urothelial carcinoma as well as UTUC. Thus, the comparison between Kim’s study and our study is difficult to make.

It is essential to follow the oncological principles and the established surgery procedure for laparoscopic surgery in urothelial carcinomas [8, 11, 14]. According to previously published papers, tumors cells may undergo retroperitoneal metastatic dissemination and dissemination along the trocar pathway under pneumoperitoneal circumstances during operation. Initial researchers despised laparoscopic operation in urothelial carcinomas because the high-pressure environment of pneumoperitoneum was thought to promote tumor dissemination and recurrence. To our best knowledge, only 12 cases of laparoscopic port-site seeding are available in English literature [23]. In our study, only one case was seen in our early experiences, which may be associated with the limited use of laparoscopic bags in the early days. Nowadays, precautionary measures have been taken into consideration to prevent potential tumor spillage. It has been stressed that direct contact between the instrument and the tumor should be forbidden during dissection. Besides, LNU must be accomplished in a closed system.

In patients with organ-confined UTUC, LNU has the advantage of minimal invasiveness and has oncological outcomes comparable to those of ONU. However, its effectiveness in patients with localized and/or locally advanced diseases remains to be proven, and the results were contradictory. Our findings were consistent with results from one single center study [10] and two recent multi-institutional studies [4, 9], which showed no independent association between surgical approach and survival, in both organ-confined and advanced UTUC patients. Unfortunately, some authors reported that relative to ONU, LNU was associated with an adverse prognosis in advanced stage patients. Fairey and colleagues [13] published a multi-institutional retrospective study comparing ONU and LNU in 849 patients. These authors report equivalent OS and CSS for the surgical approaches. However, there was a trend toward an independent association between surgical approach and RFS (OR 1.24, 95% CI 0.98–1.57, *P* = 0.08). Furthermore, when stratifying by stage on multivariate Cox regression models, LNU was independently associated with poorer RFS in patients with ≤ pT2N0 and pTanyN1-3 disease: however, there was no independent association between surgical approach and RFS in patients with pT3-4 N0 disease. In the only prospective randomized study published in the literature, Simone and colleagues [19] reported 80 UTUC patients treated with ONU (*n* = 40) and LNU (n=40). After a median follow-up of 44 months, for organ-confined disease, the two groups did not differ significantly in the rates of intravesical RFS and CSS. However, when matched for pT3 and high-grade tumors, CSS and metastasis-free survival were significantly different between the two groups, in favor of ONU.

However, the conclusions based on previous results are underpowered because of the different statistical models used. The factors of UTUC tumor location [24, 25], previous bladder tumor history [26, 27], and previous hydronephrosis [28, 29] should be included in the model because the predictive significance of these factors remains controversial. Additionally, cigarette smoking status should be included in the analysis because exposure to smoking is a significant risk factor for bladder urothelial carcinoma as well as UTUC [30]. Furthermore, imbalances are apparent in some of these important series [9, 11, 13]. The LNU group contained tumors at lower stages and they had a lower rate of LVI. These differences, were statistically significant. This may be compensated for by the multivariate analysis and further corrected using various statistical techniques; nevertheless, it reflects significant patient selection in which, generally speaking, LNU was avoided in the higher stage cases. Thus, because of the smaller proportion of higher stage cases performed laparoscopically, the overall outcome was skewed by the good prognosis of the lower stage cases. In comparison with previous results, our groups were better matched for prognostic factors, such as tumor stage, grade, and LVI, and we include some controversial elements, such as tumor location, previous bladder tumor history, and previous hydronephrosis. Therefore, we could draw more relevant conclusions. In addition, several limitations of this study should be mentioned. First, the data were collected retrospectively and reflect the experiences of two institutions. Furthermore, different bladder cuff managements were used between the LNU and ONU groups. Second, the majority of patients were underwent open procedures; moreover, the laparoscopic cohort included those operated on using retroperitoneal and transperitoneal approaches. Third, pathological specimens were not subjected to a centralized review. In additon, the follow-up period was relatively short.

## Conclusions

In summary, after a median follow-up of 60.0 months, oncological results were comparable between LNU and ONU for the treatment of localized and/or locally advanced UTUC (T_1–4_/N_0-X_). Our data could be used as evidence for equivalent cancer control outcomes between LNU and ONU in patients with T_1–4_/N_0-X_ UTUC. Further analyses, including randomized trials, are needed to generalize these conclusions to patients with more unfavorable disease characteristics.

## Abbreviations

CI: Confidence interval
CSS: Cancer-specific survival
LNU: Laparoscopic radical nephroureterectomy
LVI: Lymphovascular invasion
ONU: Radical nephroureterectomy
OR: Odds ratio
OS: Overall survival
RFS: Recurrence-free survival
RNU: Radical nephroureterectomy
UTUC: Upper tract urothelial carcinoma
WHO/ISUP: World Health Organization/International Society of Urologic Pathology
